# Supplementary material for: Co-immobilization of whole cells and enzymes by covalent organic framework for biocatalysis process intensification
Source: Nat Commun. 2024 Jun 29;15:5510. doi: 10.1038/s41467-024-49831-8 (PMC11217415; doi:10.1038/s41467-024-49831-8)
Supplement: Supplementary file 1 — Supplementary Information [file 41467_2024_49831_MOESM1_ESM.pdf]

Co-immobilization of whole cells and enzymes by covalent organic framework for biocatalysis process intensification

Zheng *et al.*

Supplementary Method 1. Synthesis of monomer

Synthesis of 2-(but-3-en-1-yloxy)-5-(2-methoxyethoxy)-terephthalohydrazide (BYTH):

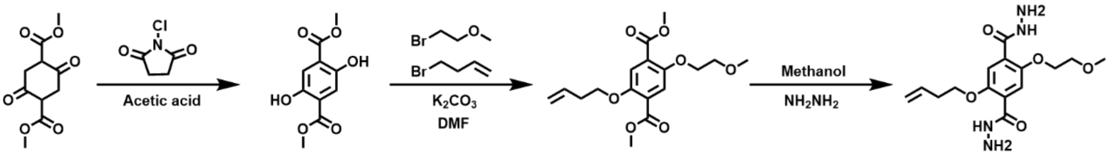

150 g of 2,5-dioxocyclohexane-1,4-dicarboxylic acid dimethyl ester and 90 g of 1-chloro-pyrrolidine-2,5-dione were added to a 1000 mL-round-bottom flask, followed with 500 mL of acetic acid. Heated the mixture to 80 °C to react for 5 h with stirring. After cooling down to room temperature, isolated the product by filtration, wash with DI water (3×100 mL) to remove unreacted acetic acid and then vacuum-dried to obtain dimethyl 2,5-dihydroxyterephthalate as a bright yellow solid. Weight: 136.8 g. Yield: 91.2%.

17 g of dimethyl 2,5-dihydroxyterephthalate and 51 g of K_2CO3 were added into a 500 mL-round-bottom flask, followed with 200 mL DMF，25 mL of 4-bromo-1-butene and 25 mL of 1-bromo-2-methoxyethane，then heated the mixture to 85 °C with stirring for 24 h. After completion, extracted the mixture with DI water and dichloromethane (3×200 mL). Remove the organic solvent via rotary evaporation and purify the residue by silica gel column chromatography to get a light yellow liquid. Weight: 16.7 g. Yield: 67.3%.

Dissolve 6 g of the obtained liquid above in 100 mL of methanol, followed with 20 mL of hydrazine hydrate (80%) and heat the mixture to reflux at 95 °C for 12 h. After the reaction was completed and cooled down to room temperature, large amount of white solid precipitated and is isolated by filtration, washed with methanol for 3 times (3×20 mL). Dry the product in a 60 °C vacuum oven to obtain a white solid. Weight: 4.9 g. Yield: 82.3%.

The synthetic procedure of 2,5-bis (2-methoxyethoxy) terephthalohydrazide (BMTH) and 2,5-bis(but-3-en-1-yloxy) terephthalohydrazide (BBTH) were similar to BYTH,

except that equal molar quantity of 1-bromo-2-methoxyethane or 4-bromo-1-butene were used instead of 4-bromo-1-butene and 1-bromo-2-methoxyethane, respectively[1,2].

Supplementary Method 2. Construction of recombinant *E. coli* for D-allulose 3-epimerase (DAE) expression

The coding sequence of DAE (GenBank: ZP_04858451) was codon-optimized for *Escherichia coli* (*E. coli)* ordered from Genewiz (Suzhou, China) as synthetic DNA, and sub-cloned into the pET21a vector (Novagen, Madison, WI, USA) between the *Nde*I and *Xho*I restriction sites. DAE was expressed in *E. coli* cells [3]. In a typical procedure, *E. coli* BL21 containing pET21a-DAE plasmid was incubated overnight at 37 °C as the preculture. The preculture was then transferred to the main culture and incubated at 37 °C until reaching OD_{600} 0.7-0.8, whereby a final concentration of 0.5 mM isopropyl β-D-1-thiogalactopyranoside (IPTG) was used to induce the expression. The cultures were shaken overnight at 22 °C, 120 rpm. After that, recombinant *E. coli* (*E. coli*/DAE, E) were harvested by centrifugation at 4 °C, 6,000 ×g, 10 min.

Supplementary Method 3. Synthesis of E@NKCOF-98

Monomer with 3.3 mg TB and 10 mg 2,5-bis (2-methoxyethoxy) terephthalohydrazide (BMTH) were dissolved in 10 mM PBS (20 mL, pH 7.4). In parallel, 40 mg *E. coli*/DAE (E, wet weight) was resuspended in the solution above. Then, 20 μL acetic acid (17.5 mM) was added to the solution. After reacting at room temperature for 2 hours, the precipitates were washed with PBS for 2 times.

Supplementary Method 4. Synthesis of E@COF-42-B

Monomer with 3.3 mg TB and 10 mg 2,5-bis(but-3-en-1-yloxy) terephthalohydrazide (BBTH) were dissolved in 10 mM PBS (20 mL, pH 7.4). In parallel, 40 mg *E. coli*/DAE (E, wet weight) was resuspended in the solution above. Then, 20 μL acetic acid (17.5 mM) was added to the solution. After reacting at room temperature for 2 hours, the precipitates were washed with PBS for 2 times.

Supplementary Method 5. Optimization of reaction conditions

The effect of pH on the activity of INU-NH_2&E-NH_2 and co-immobilized INU-NH_2&E-NH_2@NKCOF-141was analyzed at 50 °C in the pH range from 5.5 to 7.5, using 50 mM NaAc-HAc buffer (pH 5.5–6.0) and 50 mM phosphate buffer (pH 6.0–7.5). Enzymatic activity was analyzed at pH 6.5 (50 mM PB) in the temperature range from 30 to 70 °C to determine the optimum temperature. To determine the thermo-stability, the free system (INU-NH_2&E-NH_2) and co-immobilized system (INU-NH_2&E-NH_2@NKCOF-141) were incubated in PB (50 mM, pH 6.5) at different temperatures (50, 55, and 60 °C) for 90 min. The thermal stability was analyzed by determining the residual activities, which were expressed as the percentage of the initial activity. All measurements were taken in triplicate.

Supplementary Method 6. Synthesis of INU-NH_2&E-NH_2@calcium alginate

The INU-NH_2 and E-NH_2 were immobilized in calcium alginate according to the method described by Li *et al.*[4], with minor modifications as follows: the E-NH_2 (40 mg, wet weight) and 20 mg INU-NH_2 were combined with 2% (w/v) Na-alginate solution. The Na-alginate solution containing E-NH_2 and INU-NH_2 was dripped into 2% $CaCl_2$ solution (w/w), forming beads with an average diameter of 3–4 mm. The cells-enzyme particles co-immobilized by Ca-alginate were immersed in the $CaCl_2$ solution at room temperature (approximately 25 °C) for 30 minutes to solidify. Finally, the particles were washed multiple times with deionized water and stored in the refrigerator before use.

Supplementary Method 7. Synthesis of INU-NH_2&E-NH_2@polyvinyl alcohol

The INU-NH_2 and E-NH_2 were immobilized in polyvinyl alcohol (PVA) according to the method described by Kao *et al.*[5], with minor modifications as follows: the mixture, consisting of E-NH_2 (40 mg, wet weight) and 20 mg INU-NH_2, was thoroughly combined with an equal volume of polyvinyl alcohol (14% w/w). The PVA-cell-enzyme mixture was carefully added into a saturated boric acid solution and stirred gently for

30-40 min to create spherical beads. The fragile beads formed in this manner were subsequently transferred to a 0.5 M sodium phosphate solution for 0.5-1 hour for gel hardening. Finally, the INU-NH_2&E-NH_2@polyvinyl alcohol was washed multiple times with deionized water and stored in the refrigerator before use.

Supplementary Method 8. Synthesis of INU-NH_2&E-NH_2@ZIF-8

The INU-NH_2 and E-NH_2 were immobilized in ZIF-8 according to the method described by Liang et al.[6], with minor modifications as follows: the E-NH_2 (40 mg, wet weight) and INU-NH_2 (2.0 mL, 10.0 mg mL^{-1} in deionized water) were suspended in an aqueous solution containing 2-methylimidazole, (160 mM, 25 mL), followed by the addition of a zinc acetate dihydrate aqueous solution (40 mM, 25 mL) while stirring at 200 rpm. After 30 min, the INU-NH_2&E-NH_2@ZIF-8 was washed three times with deionized water to eliminate excess ZIF-8 precursors.

Supplementary Method 9. Construction of recombinant *E. coli* for *Lipase* expression

The coding sequence of *Lipase* (GenBank: KR996514) was codon-optimized for *Escherichia coli* (*E. coli)* ordered from Genewiz (Suzhou, China) as synthetic DNA, and sub-cloned into the pET28a vector (Novagen, Madison, WI, USA) between the *Nco* I and *Hind* III restriction sites. *Lipase* in *E. coli* cells was expressed [7]. In a typical procedure, *E. coli* BL21 containing pET28a-*Lipase* plasmid was incubated overnight at 37 °C as the preculture. The preculture was then transferred to the main culture and incubated at 37 °C until reaching OD_{600} 0.6-0.8, whereby a final concentration of 1 mM IPTG was used to induce the expression. The cultures were shaken overnight at 22 °C, 120 rpm. After that, recombinant *E. coli* (*E. coli*/*Lipase*, E/*Lipase*) were harvested by centrifugation at 4 °C, 6000 ×g, 10 min.

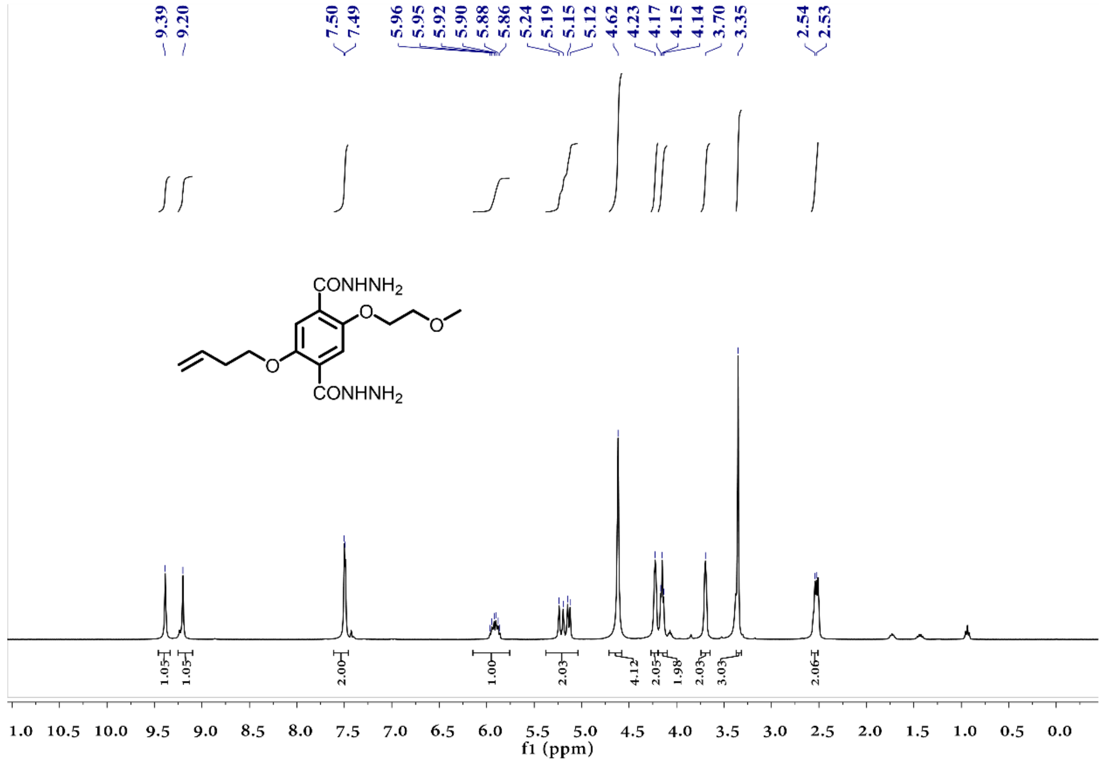

Supplementary Figure 1. ^{1}H NMR spectrum of 2-(but-3-en-1-yloxy)-5-(2-methoxyethoxy)-terephthalohydrazide (BYTH).

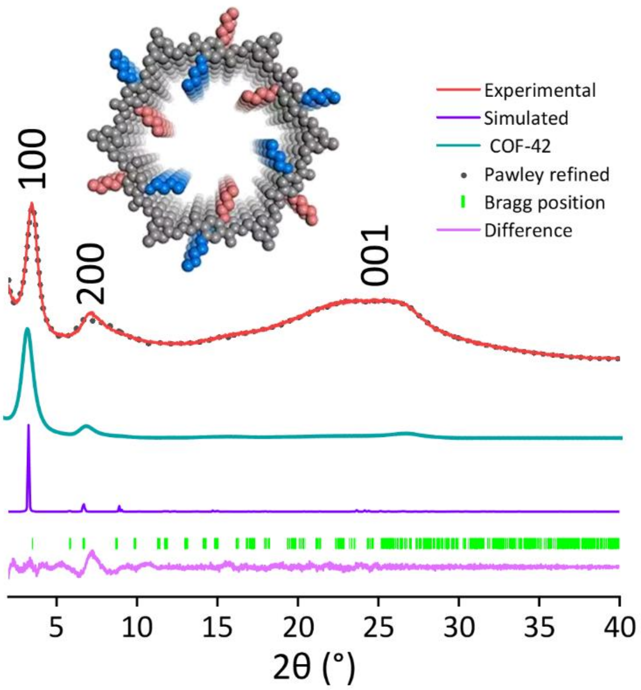

Supplementary Figure 2. Pawley refinement against the PXRD pattern of NKCOF-141.

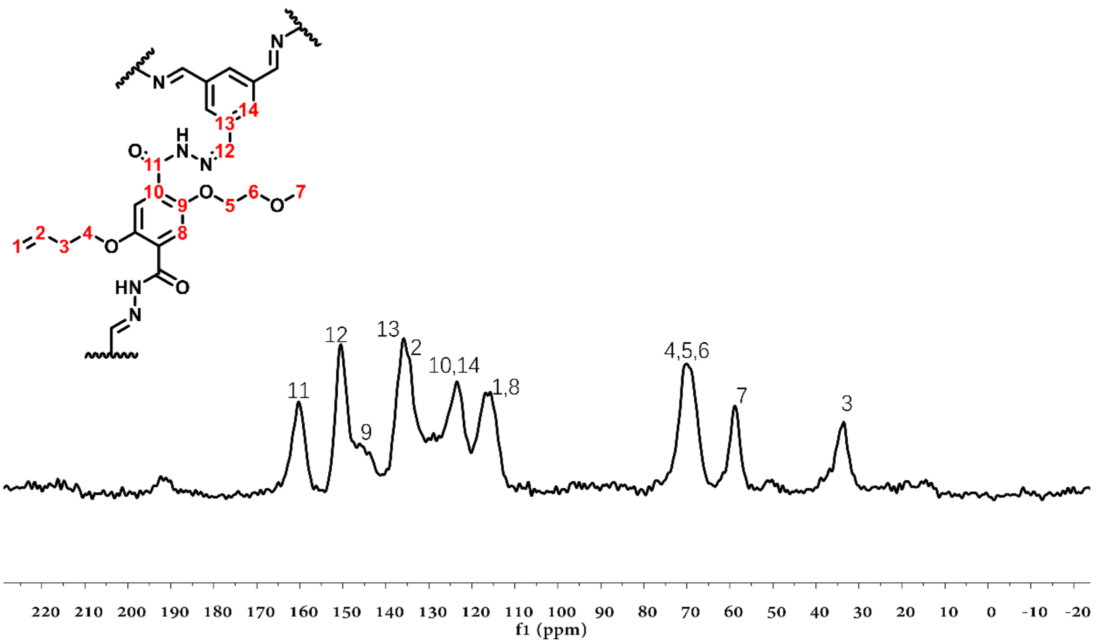

Supplementary Figure 3. Solid-state ^{13}C NMR spectra of NKCOF-141.

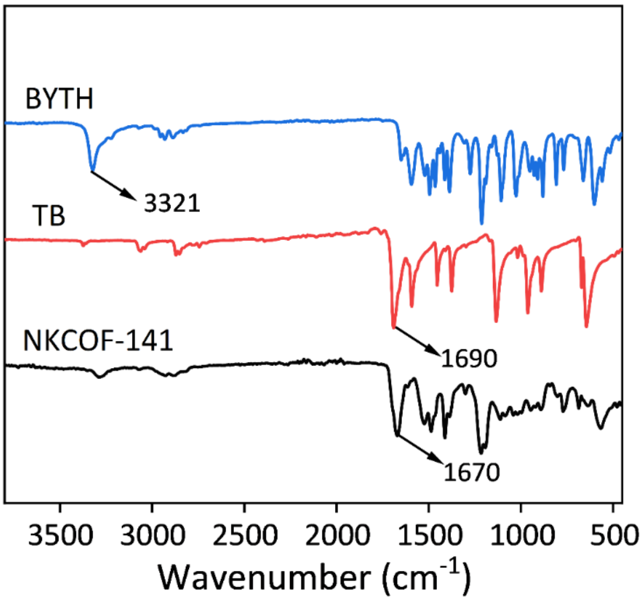

Supplementary Figure 4. Fourier transform infrared (FT-IR) spectra of BYTH, TB and NKCOF-141.

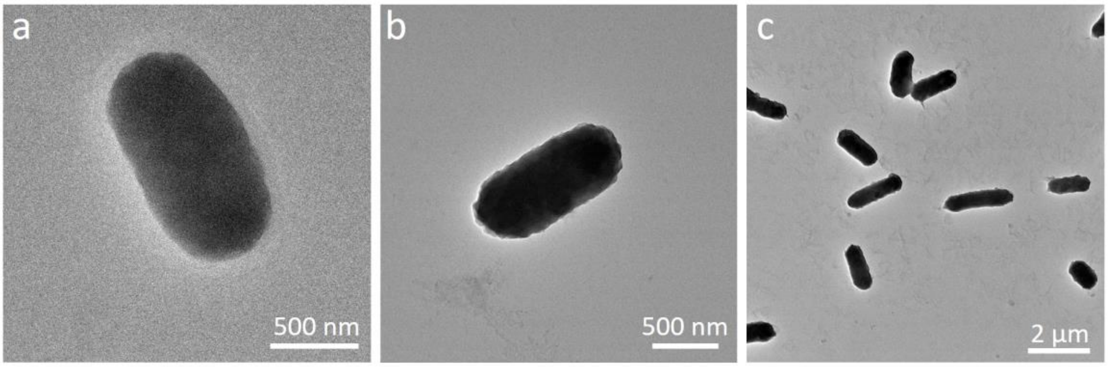

Supplementary Figure 5. Transmission electron microscope (TEM) images of cells. (**a**) Free *E. coli*. (**b**) E@NKCOF-141. (**c**) E@NKCOF-141 under low magnification. Three independent experiments were repeated with similar results (n=3).

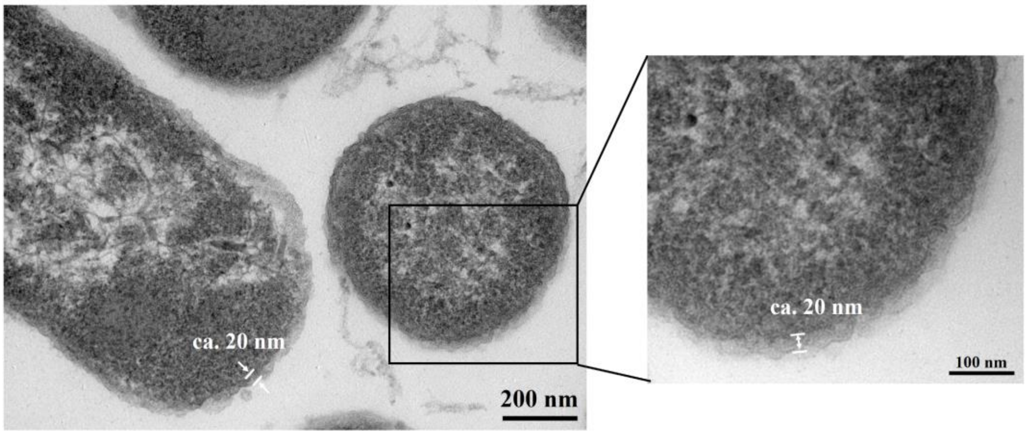

Supplementary Figure 6. TEM micrographs of microtomed E@NKCOF-141 at different magnifications. Three independent experiments were repeated with similar results (n=3).

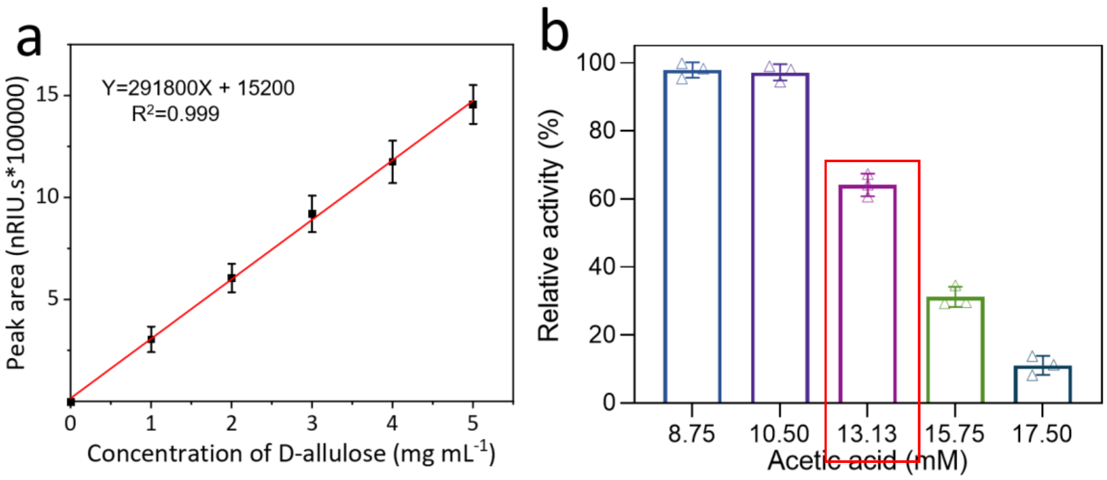

Supplementary Figure 7. The viability assays of *E. coli* cells expressing DAE. (**a**) Standard curve of D-allulose analyzed by HPLC. (**b**) The activity of E@NKCOF-141 synthesized at different acetic acid concentrations. Error bars mean ± s.d. received from three independent experiments (n=3). Source data are provided as a Source Data file.

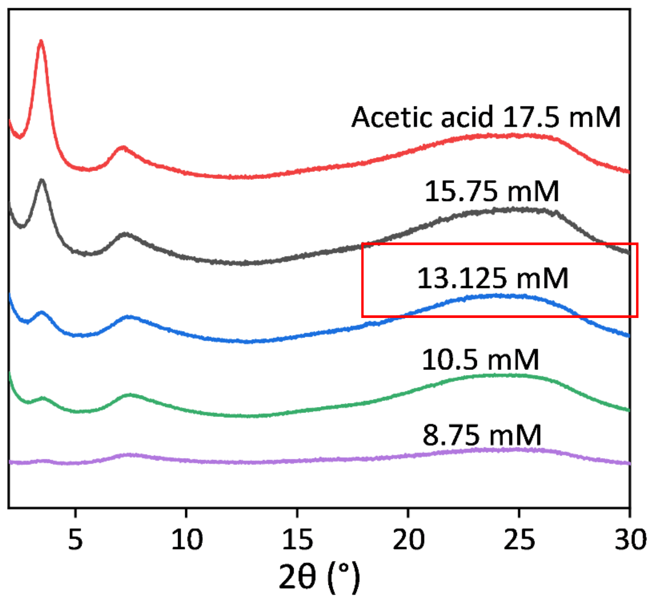

Supplementary Figure 8. PXRD patterns of E@NKCOF-141 synthesized at different acetic acid concentrations.

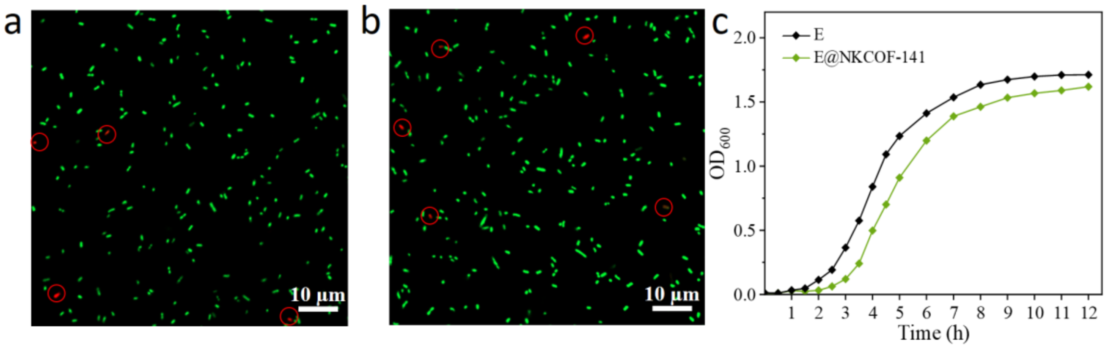

Supplementary Figure 9. Evaluation of the biocompatibility of the immobilization process. Live/dead assay for (**a**) free *E. coli* cells and (**b**) E@NKCOF-141, green - live cells, red - dead cells. Three independent experiments were repeated with similar results (n=3). (**c**) Growth curve of native E (black curve) and E@NKCOF-141 (green curve).

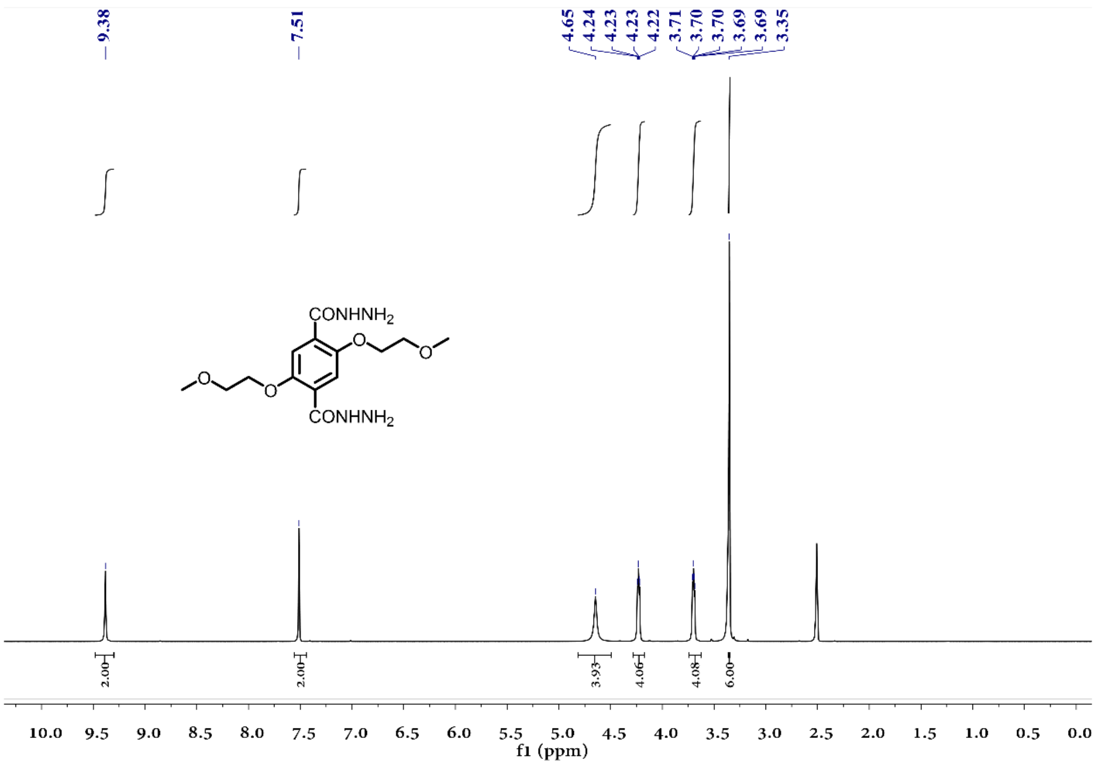

Supplementary Figure 10. ^{1}H NMR spectrum of 2,5-bis (2-methoxyethoxy) terephthalohydrazide (BMTH).

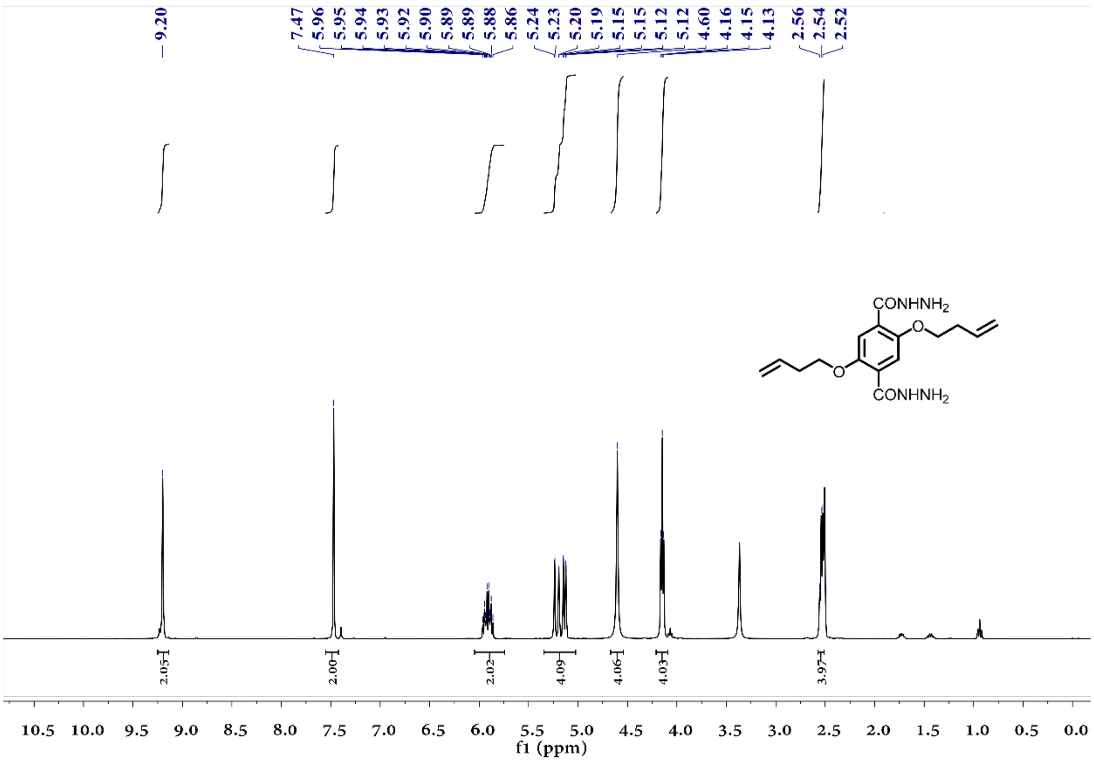

Supplementary Figure 11. ^{1}H NMR spectrum of 2,5-bis(but-3-en-1-yloxy) terephthalohydrazide (BBTH).

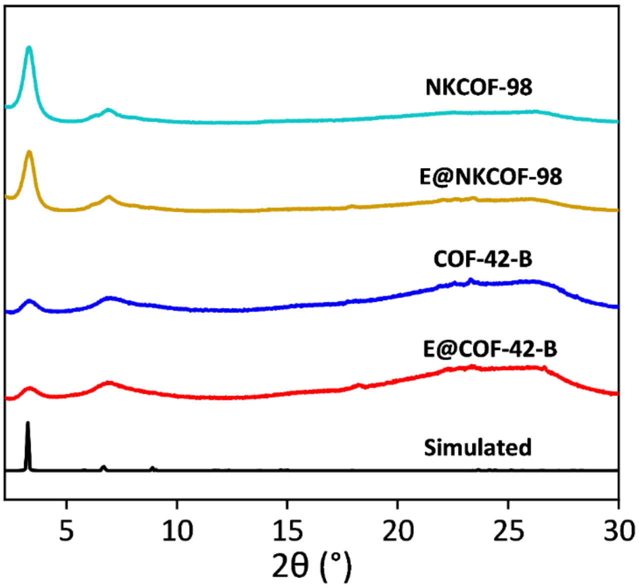

Supplementary Figure 12. PXRD patterns of NKCOF-98 and COF-42-B before and after immobilized *E. coli* (E).

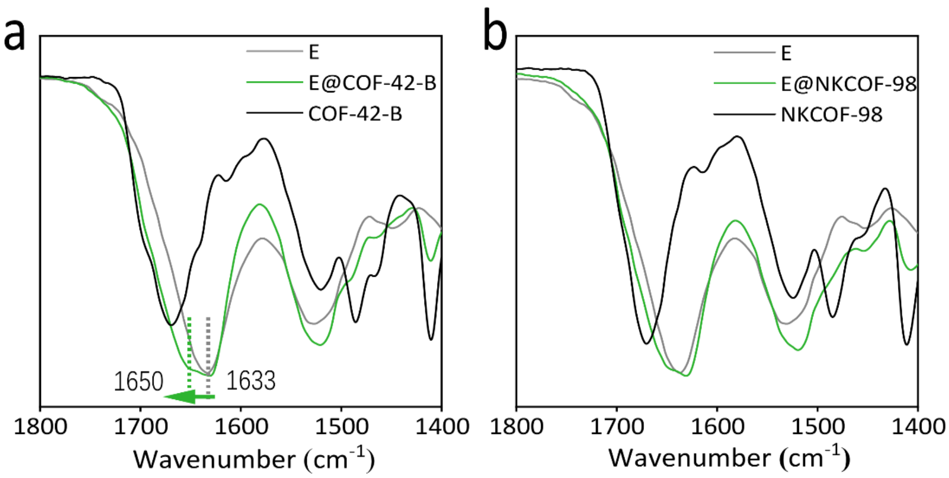

Supplementary Figure 13. FT-IR spectra of E@COFs. FT-IR spectra of the (**a**) COF-42-B and (**b**) NKCOF-98 before and after immobilized *E. coli* (E).

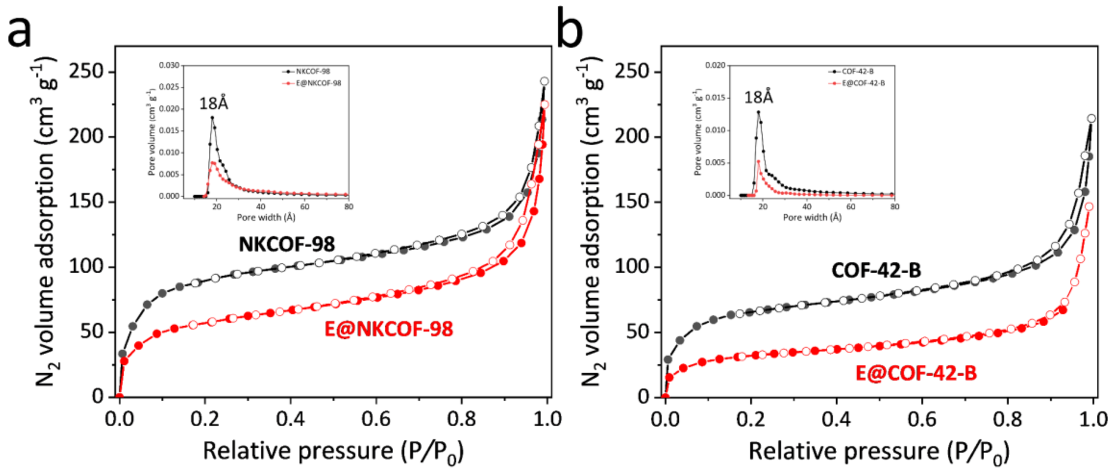

Supplementary Figure 14. N_2 sorption isotherms of E@COFs. N_2 sorption isotherms of (**a**) NKCOF-98 and E@NKCOF-98, (**b**) COF-42-B and E@COF-42-B and the corresponding pore size distribution. The curve consisting of hollow points represents the desorption isotherm, while the curve consisting of solid points represents the adsorption isotherm.

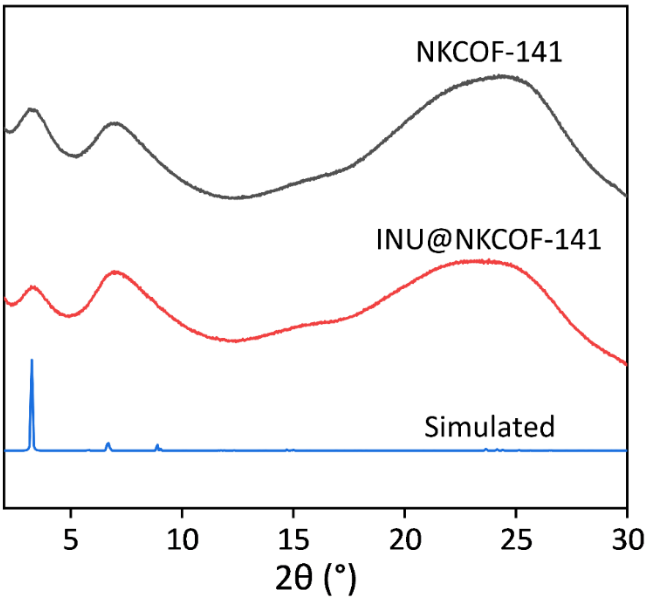

Supplementary Figure 15. PXRD patterns of NKCOF-141 and INU@NKCOF-141.

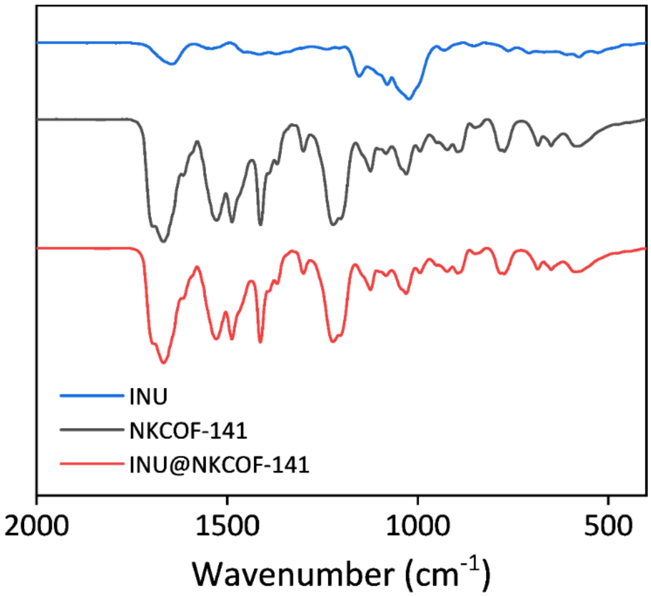

Supplementary Figure 16. FT-IR spectra of INU before and after NKCOF-141 immobilization.

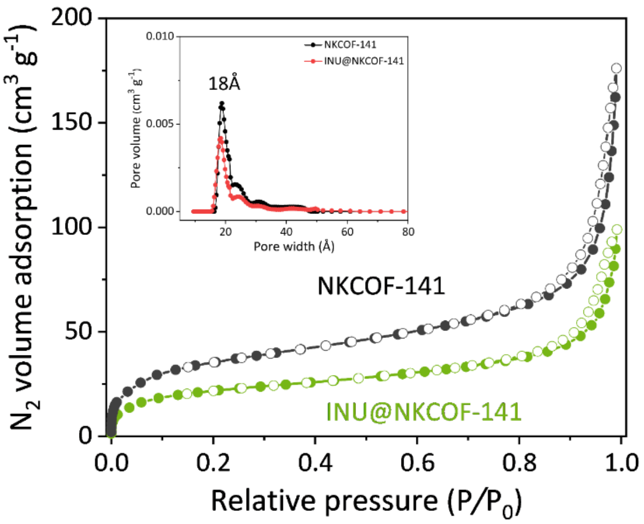

Supplementary Figure 17. N_2 sorption isotherms of NKCOF-141 and INU@NKCOF-141, and the corresponding pore size distribution.

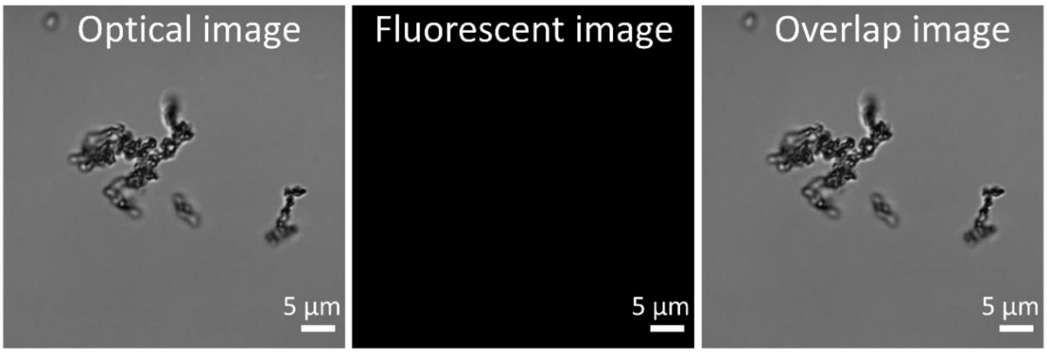

Supplementary Figure 18. CLSM images of NKCOF-141 incubated in PBS solution containing FITC-INU. Three independent experiments were repeated with similar results (n=3).

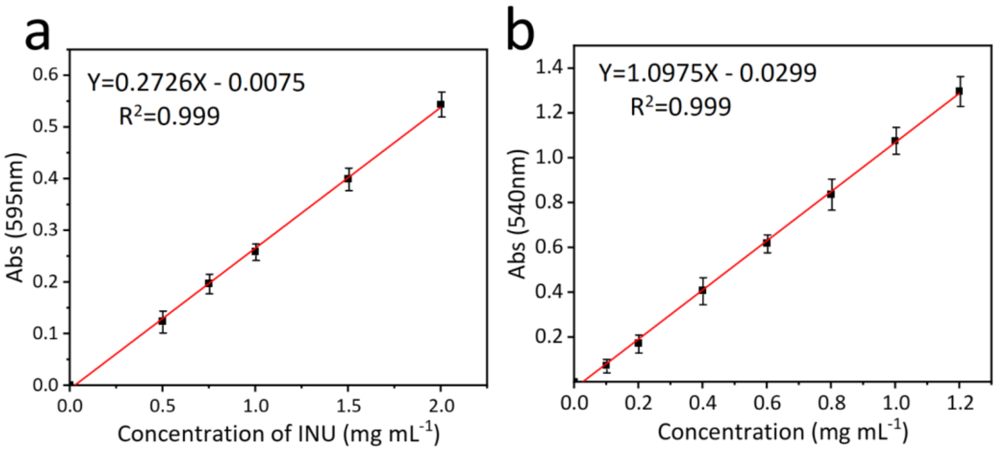

Supplementary Figure 19. The standard curve. (**a**) Corresponding standard curve of INU based on Bradford assay. (**b**) Standard curve of reducing sugar measured by DNS method.

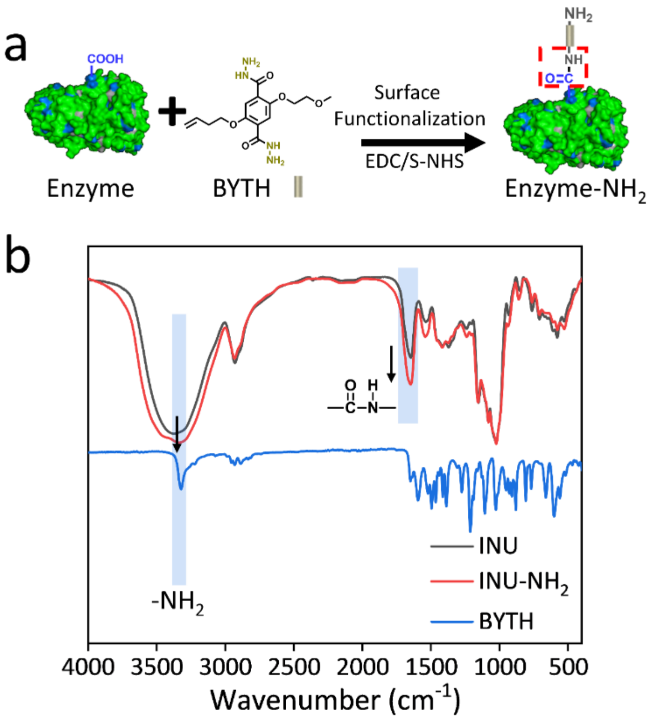

Supplementary Figure 20. Pretreatment of enzyme with BYTH. (**a**) Schematic illustration of inulinase (INU) modified by BYTH. (**b**) FT-IR spectra of inulinase (INU) before and after BYTH modification.

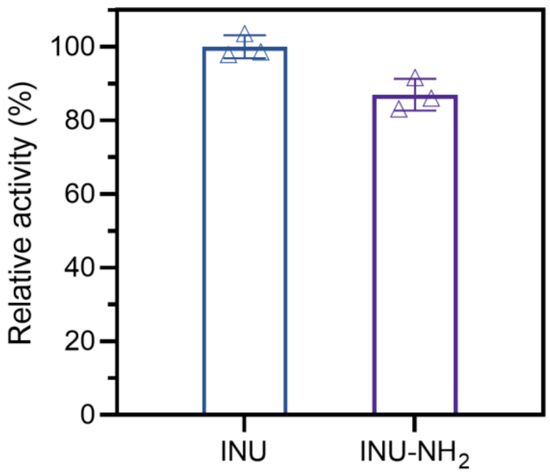

Supplementary Figure 21. Activity analysis of INU before and after BYTH modification. Error bars mean ± s.d. received from three independent experiments. Source data are provided as a Source Data file.

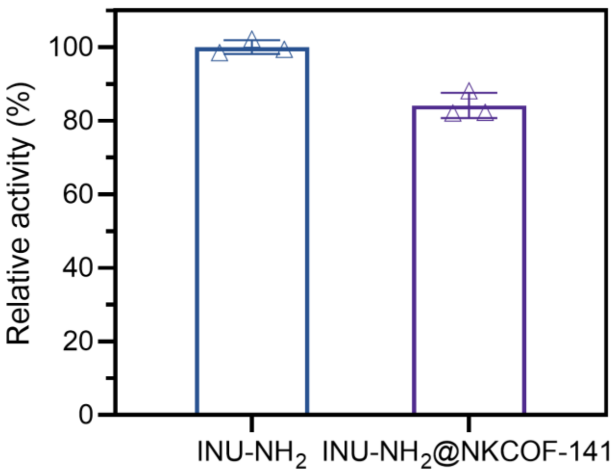

Supplementary Figure 22. Activity analysis of INU-NH2 and INU-NH2@NKCOF-141. Error bars mean ± s.d. received from three independent experiments. Source data are provided as a Source Data file.

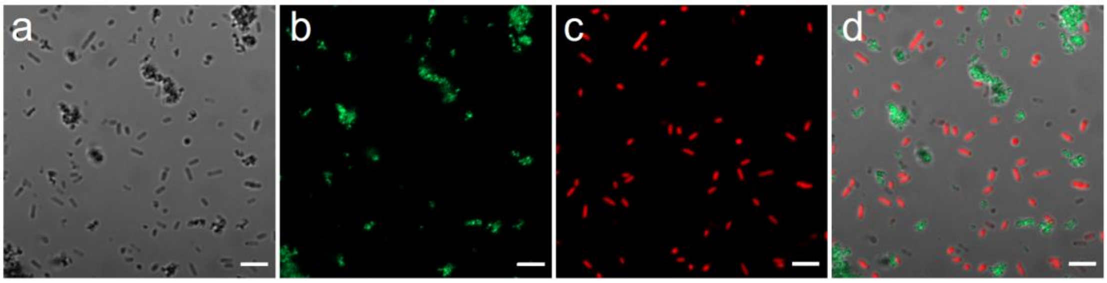

Supplementary Figure 23. CLSM images of FITC-INU-NH_2&E@NKCOF-141. (**a**) Optical image. (**b**) Excitation at 488 nm and tracking the fluorescence within a spectral range of 480 to 540 nm. (**c**) Excitation at 561 nm and tracking the fluorescence within a spectral range of 580 to 680 nm. (**d**) Overlap image of three images. Scale bar: 5 µm. The cell was stained with propidium iodide (red). Three independent experiments were repeated with similar results (n=3).

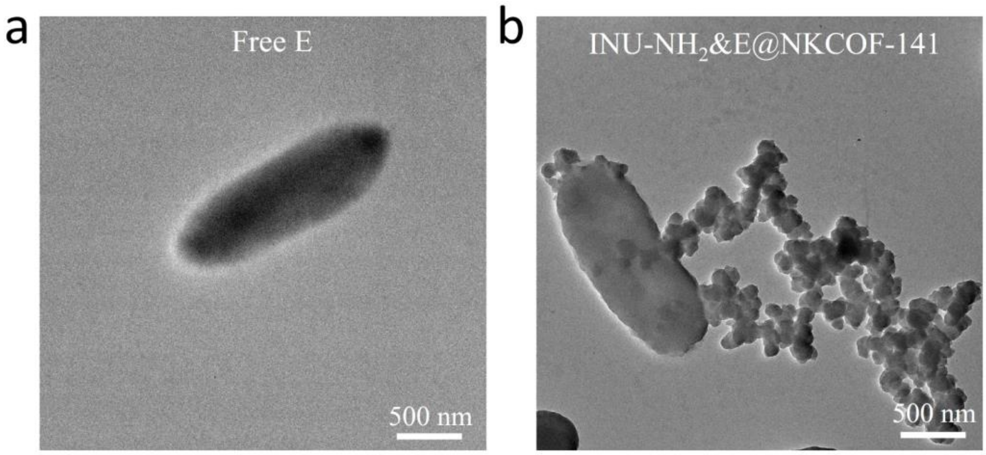

Supplementary Figure 24. TEM images of enzyme and cell co-immobilization. TEM images of (**a**) free E and (**b**) INU-NH_2&E@NKCOF-141. Three independent experiments were repeated with similar results (n=3).

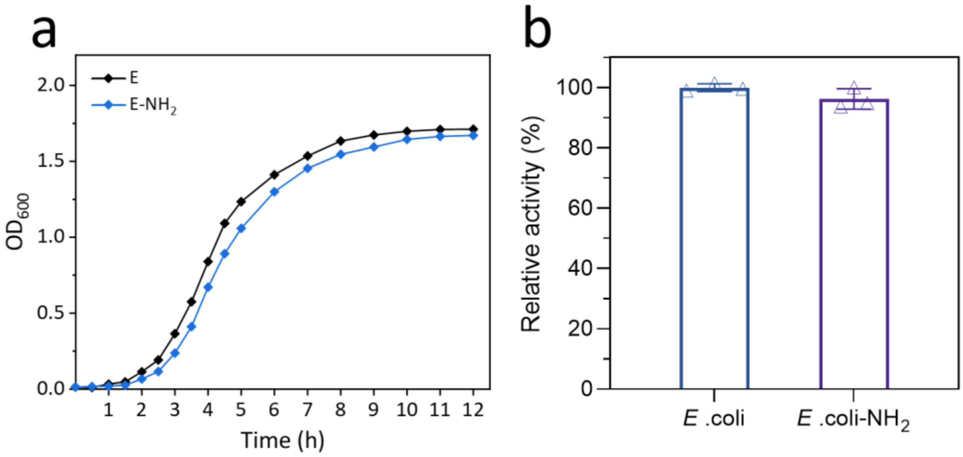

Supplementary Figure 25. Viability of cells after pretreatment with BYTH. The growth curve (**a**) and activity analysis (**b**) of *E. coli* before and after BYTH modification. Error bars mean ± s.d. received from three independent experiments. Source data are provided as a Source Data file.

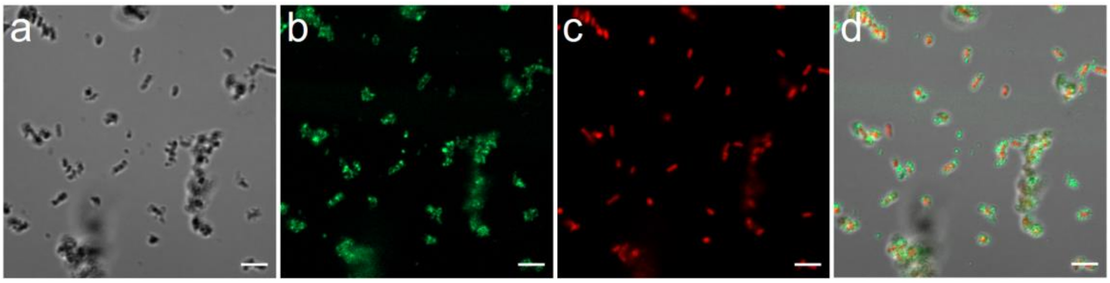

Supplementary Figure 26. CLSM images of FITC-INU-NH_2&E-NH_2@NKCOF-141. (**a**) Optical image. (**b**) Excitation at 488 nm and tracking the fluorescence within a spectral range of 480 to 540 nm. (**c**) Excitation at 561 nm and tracking the fluorescence within a spectral range of 580 to 680 nm. (**d**) Overlap image of three images. Scale bar: 5 µm. The cell was stained with propidium iodide (red). Three independent experiments were repeated with similar results (n=3).

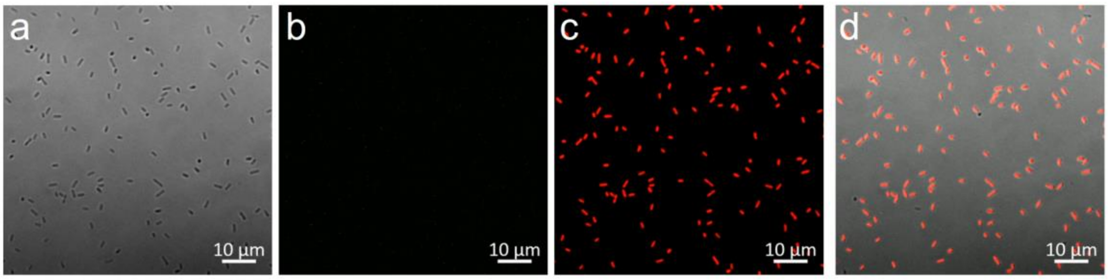

Supplementary Figure 27. CLSM images of enzymes and cellular reactions without COF. (**a**) Optical image. (**b**) Excitation at 488 nm and tracking the fluorescence within a spectral range of 480 to 540 nm. (**c**) Excitation at 561 nm and tracking the fluorescence within a spectral range of 580 to 680 nm. (**d**) Overlap image of three images. The cell was stained with propidium iodide (red). Three independent experiments were repeated with similar results (n=3).

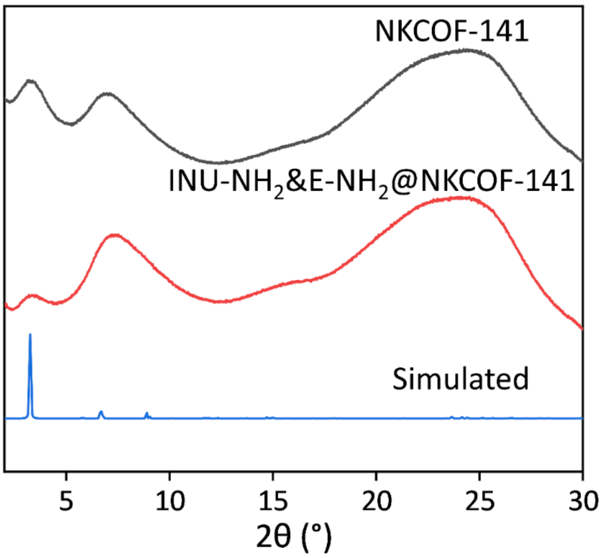

Supplementary Figure 28. PXRD patterns of NKCOF-141 and INU-NH_2&E-NH_2@NKCOF-141.

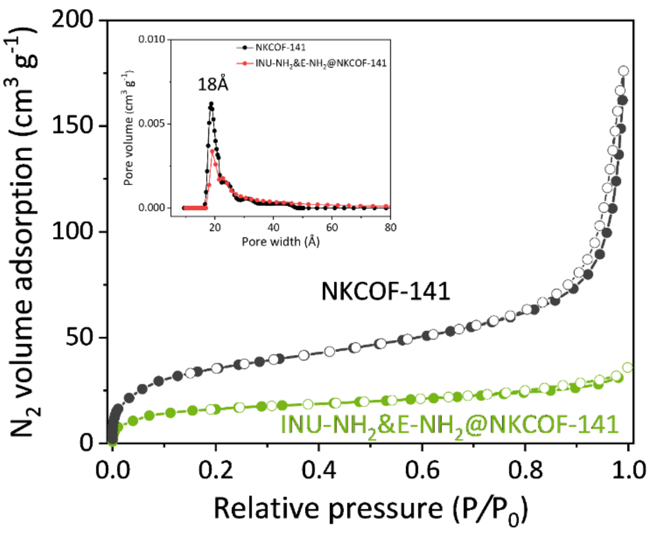

Supplementary Figure 29. N_2 sorption isotherms of NKCOF-141 and INU-NH_2&E-NH_2@NKCOF-141, and the corresponding pore size distribution.

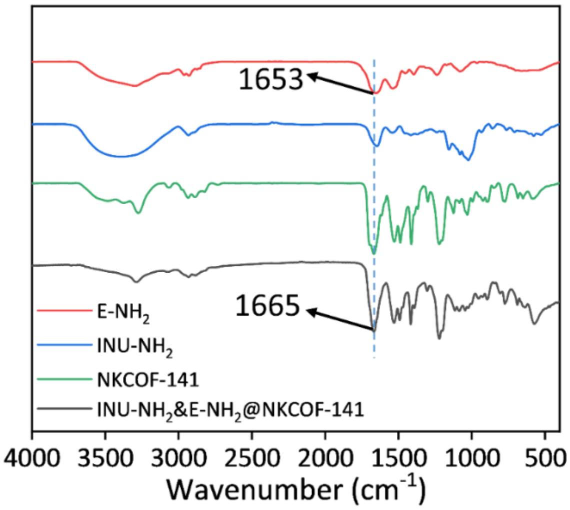

Supplementary Figure 30. FT-IR spectra of E-NH_2, INU-NH_2, NKCOF-141 and INU-NH_2&E-NH_2@NKCOF-141.

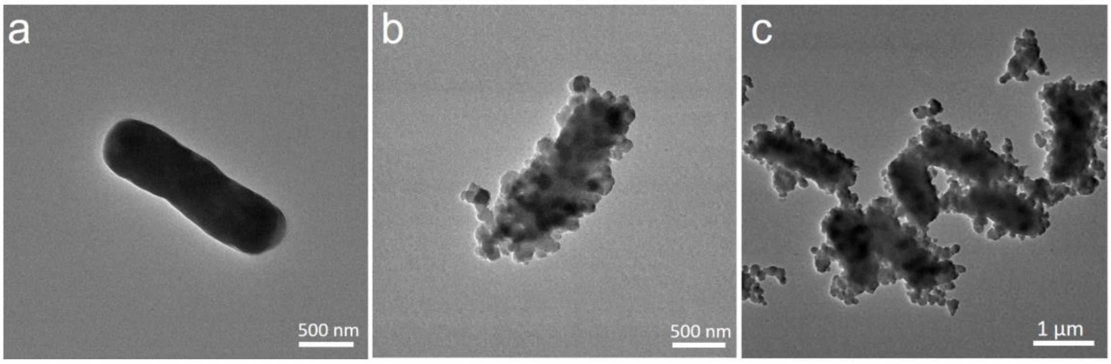

Supplementary Figure 31. TEM images of INU-NH_2 and E-NH_2 co-immobilization. (**a**) E-NH_2. (**b**) INU-NH_2&E-NH_2@NKCOF-141. (**c**) INU-NH_2&E-NH_2@NKCOF-141 under low magnification. Three independent experiments were repeated with similar results (n=3).

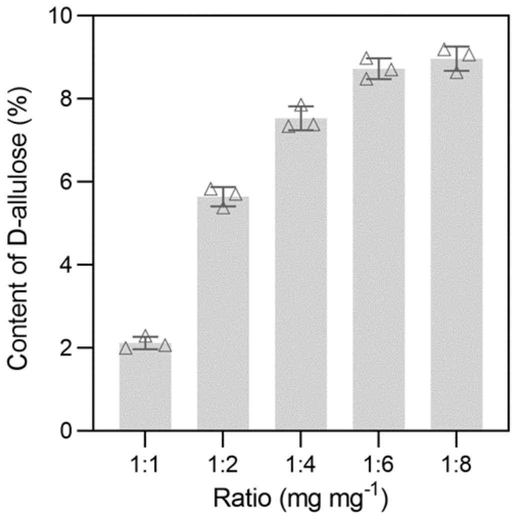

Supplementary Figure 32. The content of D-allulose with different ratios INU-NH_2/E-NH_2 in INU-NH_2&E-NH_2@NKCOF-141 using 10 g/L inulin as substrate at 50 °C in 10 min. Error bars mean ± s.d. received from three independent experiments. The ratio of INU-NH_2 and E-NH_2 in the co-immobilization system was regulated to optimize the production of D-allulose. When the ratio was increased above 1:4 (mg mg^{-1}), the content of D-allulose showed no significant increase. Therefore, the 1:4 ratio was chosen as the optimal ratio for co-immobilization. Source data are provided as a Source Data file.

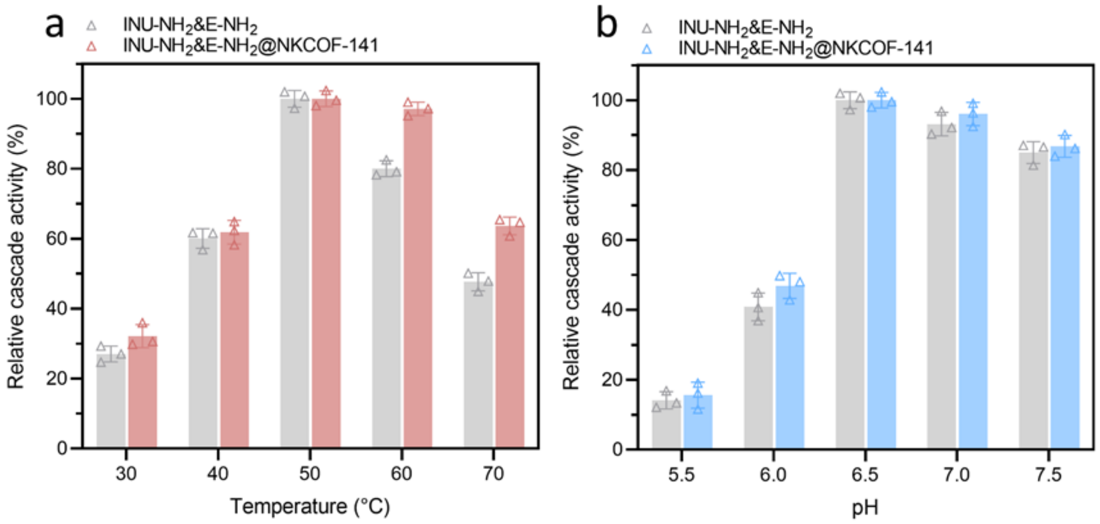

Supplementary Figure 33. The cascade activity of free INU-NH_2&E-NH_2 and co-immobilized system. (**a**) Effect of temperature on the activities of INU-NH_2&E-NH_2 and INU-NH_2&E-NH_2@NKCOF-141. The optimal temperature was determined using the standard activity assay at various temperatures ranging from 30 °C to 70 °C and pH 6.5. (**d**) Effect of pH on the activities of INU-NH_2&E-NH_2 and INU-NH_2&E-NH_2@NKCOF-141. The optimal pH was determined using the standard activity assay over a pH range of 5.5-7.5 at 50 °C. Error bars mean ± s.d. received from three independent experiments. Source data are provided as a Source Data file.

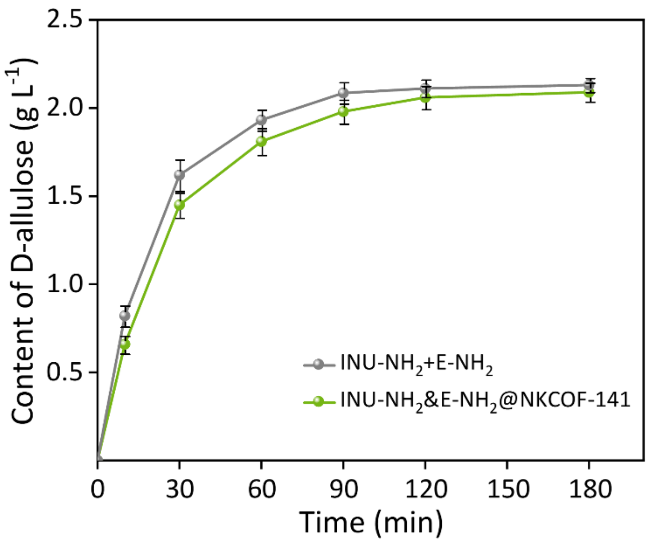

Supplementary Figure 34. The kinetic profiles of the content of D-allulose in INU-NH_2&E-NH_2 before and after NKCOF-141 immobilization. Error bars mean ± s.d. received from three independent experiments.

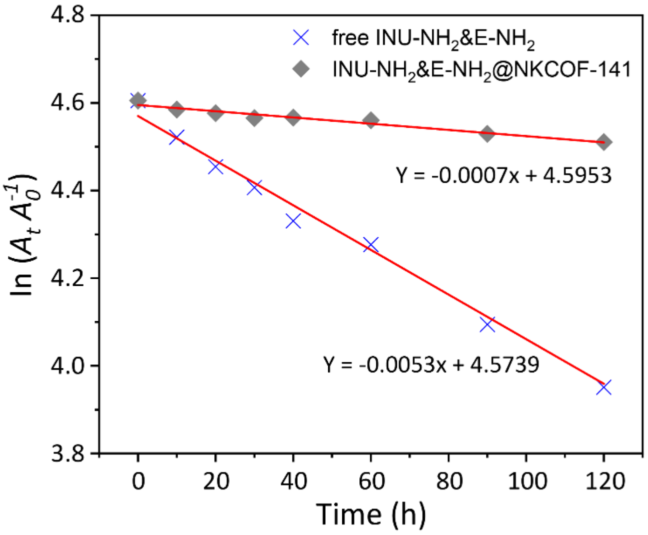

Supplementary Figure 35. Thermal kinetics profile of free and co-immobilization of INU-NH_2&E-NH_2.

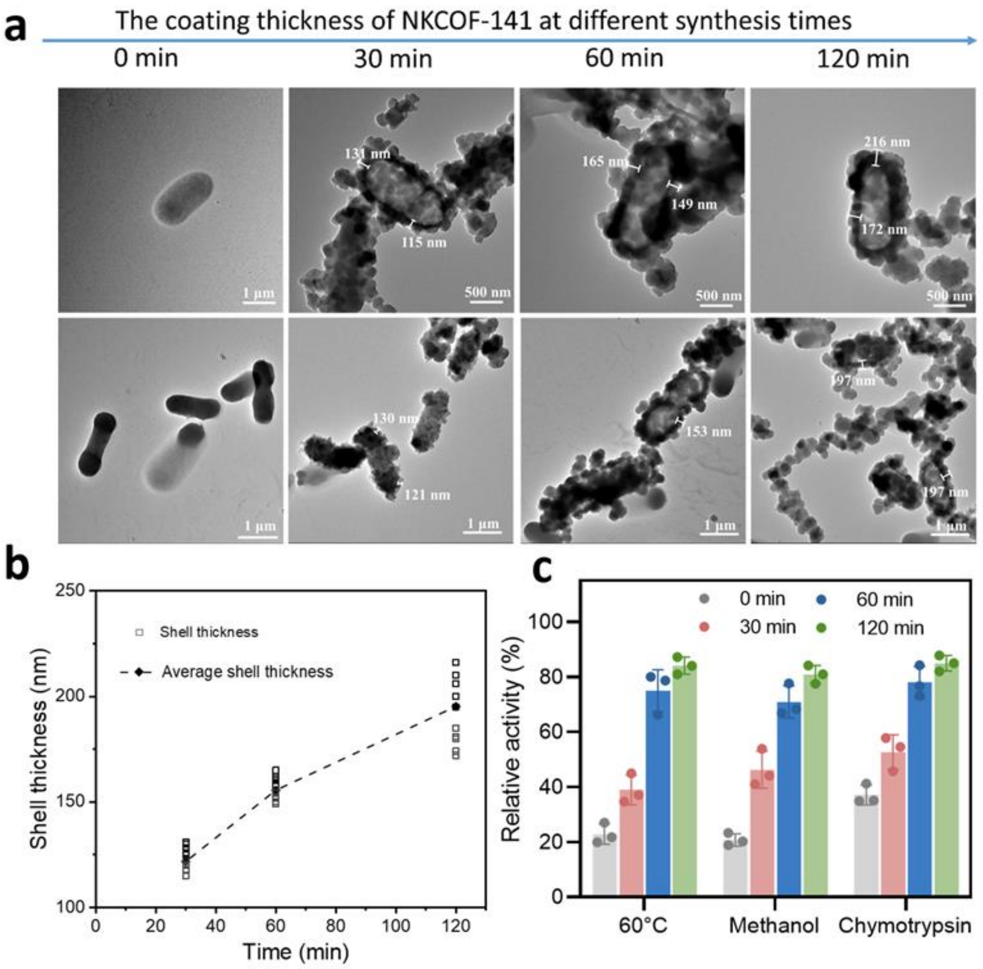

Supplementary Figure 36. Thickness modulation and protective efficiency with COF coating. (**a**) The images of co-immobilization under different synthesis times. Three independent experiments were repeated with similar results (n=3). (**b**) Measurements of COF coating thickness at 30, 60 and 120 minute times. (**c**) The protective efficiencies of synthesized INU-NH_2&E-NH_2@NKCOF-141 at different times. Error bars mean ± s.d. received from three independent experiments. Source data are provided as a Source Data file.

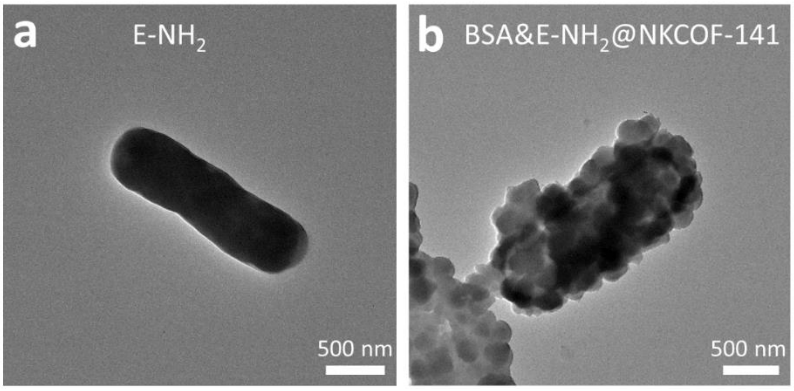

Supplementary Figure 37. TEM images of BSA and E-NH_2 co-immobilization. (**a**) E-NH_2. (**b**) BSA&E-NH_2@NKCOF-141. Three independent experiments were repeated with similar results (n=3).

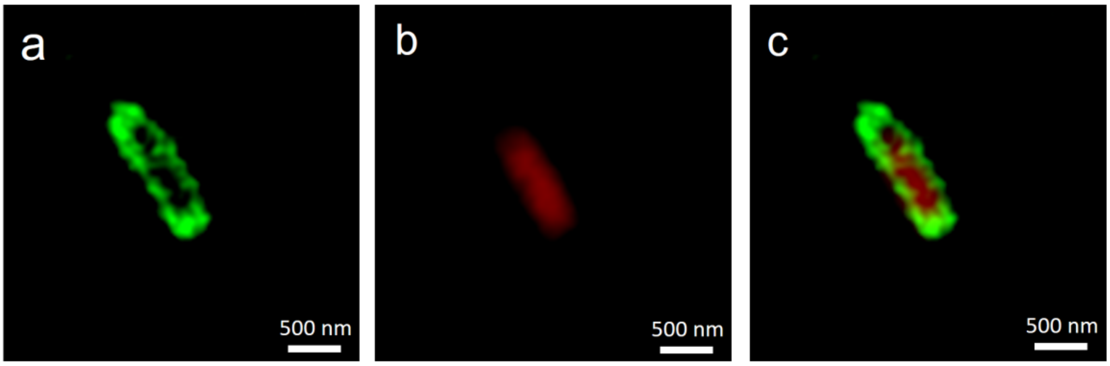

Supplementary Figure 38. CLSM images of FITC-BSA&E-NH_2@NKCOF-141. (**a**) Excitation at 488 nm and tracking the fluorescence within a spectral range of 480 to 540 nm. (**b**) Excitation at 561 nm and monitoring the fluorescence within a spectral range of 580 to 680 nm. (**c**) Overlap image of two images. The cell was stained with propidium iodide (red). Three independent experiments were repeated with similar results (n=3).

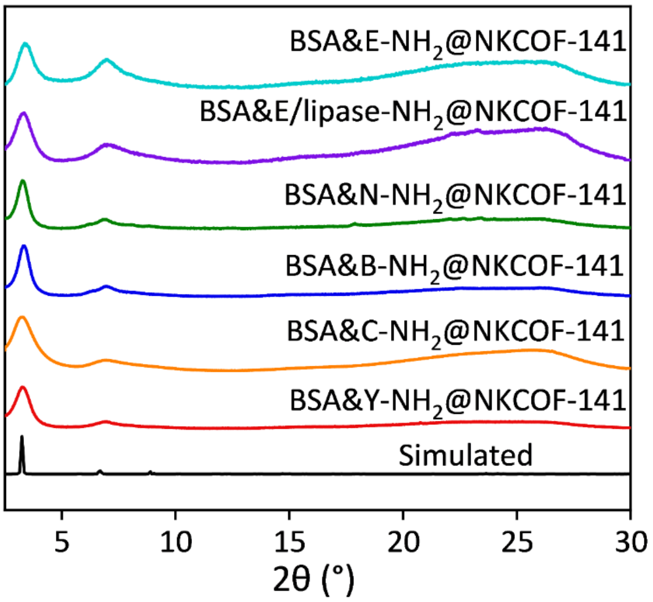

Supplementary Figure 39. PXRD patterns of BSA&E-NH_2@NKCOF-141, BSA&E/lipase-NH_2@NKCOF-141, BSA&N-NH_2@NKCOF-141, BSA&B-NH_2@NKCOF-141, BSA&C-NH_2@NKCOF-141 and BSA&Y-NH_2@NKCOF-141.

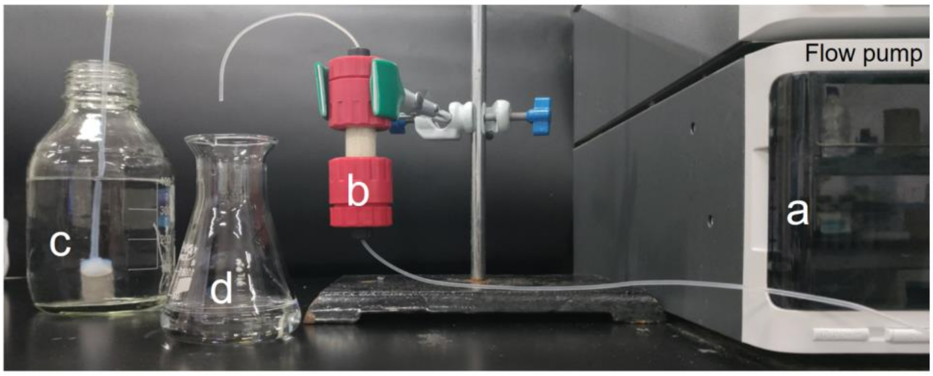

Supplementary Figure 40. Schematic diagram of a continuous flow reaction unit.
(**a**) Flow pump. (**b**) Reactor column. (**c**) Substrate. (**d**) Effusion.

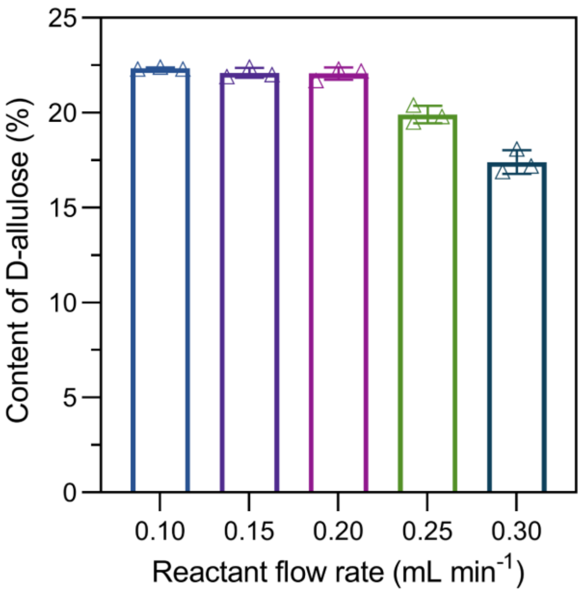

Supplementary Figure 41. The effect of flow rate on the content of D-allulose at 50 °C. Error bars mean ± s.d. received from three independent experiments. Source data are provided as a Source Data file.

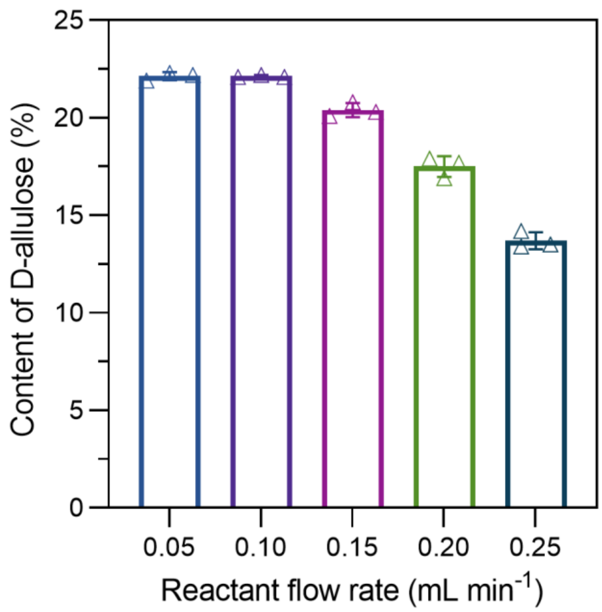

Supplementary Figure 42. The effect of flow rate on the content of D-allulose at room temperature (30 °C). Error bars mean ± s.d. received from three independent experiments. Source data are provided as a Source Data file.

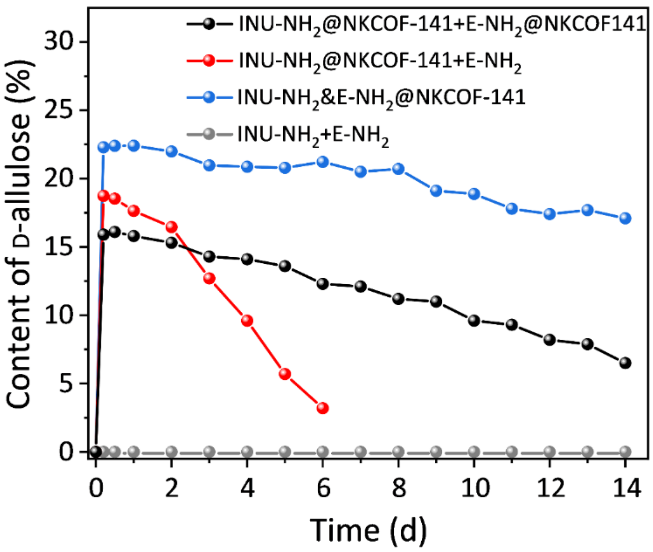

Supplementary Figure 43. Time-dependent content for D-allulose with different immobilization methods.

Supplementary Table 1. Amino acid composition analysis of exoinulinase from *Aspergillus niger* (GenBank: BAD01476.1).

Amino acid	Number	% mol/mol
**Arginase**	14	2.61
**Lysine**	17	3.17
**Aspartic acid**	31	5.77
**Glutamic acid**	18	3.35

Supplementary Table 2. Half-life ($t_{1/2}$) and thermal deactivation constant (k_d) of free and co-immobilized system.

Temp °C	k_d min^{-1}		$t_{1/2}$ (min)	
	Free	co-immobilization	Free	co-immobilization
50	0.0053	0.0007	131	990

Supplementary Table 3. The production of D-allulose from inulin.

Name of catalysts	STY of D-allulose	Operational stability	Ref
Inulinase (INU) from *Aspergillus niger*<br>D-Psicose 3-epimerase (3PE) from *Agrobacterium tumefaciens*<br>**INU + 3PE**	—	—	8
A. piperis exoinulinase and *Dorea sp.* DAEase<br>**exoinulinase+DAE**	—	—	9
Bacillus velezensis (BvInu), and DAE from *Ruminococcus sp.*<br>**BvInu +DAE**	—	—	10
ENINU and EXINU from *Pseudomonas mucidolens*<br>DAE from *Agrobacterium fabrum*<br>**NGCacher_ENINU_CsgA/NGTag_EXINU/DAERK**	—	—	11
CSCA from *Bacillus amyloliquefaciens* NB<br>DAE from *Agrobacterium tumefaciens* ATCC33970<br>Catechol-modified alginate with titanium ions (Alg(Ti)PDA)<br>**Alg(Ti)PDA immobilized CSCA-linker-DAE**	59.4 g L^{-1} day^{-1}	After 24 cycles for 8 days, >80% of initial activities.	12
Inulinas (INU) from *Aspergillus niger*<br>*E. coli*/DAE (DAE from *Ruminococcus* sp.)<br>**INU-NH_2&E-NH_2@NKCOF-141**	161.28 g L^{-1} day^{-1}	>80% of initial productivity (129 g L^{-1} day^{-1}) within 0-24 hours	**This work**
	80.64 g L^{-1} day^{-1}	Remained at 76% of initial productivity (61.56 g L^{-1} day^{-1}) after continuous conversion for 14 days	

Supplementary references

1. Zheng, Y. et al. Green and scalable *in-situ* fabrication of high-performance biocatalysts using covalent organic frameworks as enzyme carriers. *Angew. Chem. Int. Ed.* **61**, e202208744 (2022).

2. Li, M. et al. Fabricating covalent organic framework capsules with commodious microenvironment for enzymes. *J. Am. Chem. Soc.* **142**, 6675-6681 (2020).

3. Zhu, Y. et al. Overexpression of D-psicose 3-epimerase from *Ruminococcus* sp. in *Escherichia coli* and its potential application in D-psicose production. *Biotechnol. Lett.* **34**, 1901-1906 (2012).

4. Li, Y., Wang, Z., Xu, X. & Jin, L. A ca-alginate particle co-immobilized with *Phanerochaete chrysosporium* cells and the combined cross-linked enzyme aggregates from *Trametes versicolor*. *Bioresource Technol.* **198**, 464-469 (2015).

5. Kao, W., Wu, J., Chang, C. & Chang, J. Cadmium biosorption by polyvinyl alcohol immobilized recombinant *Escherichia coli*. *J. Hazard. Mater.* **169**, 651-658 (2009).

6. Liang, K. et al. Biomimetic mineralization of metal-organic frameworks as protective coatings for biomacromolecules. *Nat. Commun.* **6**, 7240 (2015).

7. Su, H. et al. Cloning, expression, and characterization of a cold-active and organic solvent-tolerant lipase from *Aeromicrobium* sp. SCSIO 25071. *J. Microbiol. Biotechnol.* **26**, 1067-1076 (2016).

8. Song, Y., Oh, C. & Bae, H. J. Simultaneous production of bioethanol and value-added D-psicose from Jerusalem artichoke (*Helianthus tuberosus* L.) tubers. *Bioresource Technol.* **244**, 1068-1072 (2017).

9. Li, W. et al. One-pot production of D-allulose from inulin by a novel identified thermostable exoinulinase from *Aspergillus piperis* and *Dorea* sp. D-allulose 3-epimerase. *Process Biochem.* **99**, 87-95 (2020).

10. Zhu, P. et al. A one-pot two-enzyme system on the production of high value-added D-allulose from Jerusalem artichoke tubers. *Process Biochem.* **88**, 90-96 (2020).

11. Chen, Y., Chen, Y., Ming, D., Zhu, L. & Jiang, L. Highly efficiency production of D-allulose from inulin using curli fiber multi-enzyme cascade catalysis. *Int. J. Biol. Macromol.* **241**, 124468 (2023).

12. Huang, Y. et al. Fusion and secretory expression of an exo-inulinase and a D-allulose 3-epimerase to produce D-allulose syrup from inulin. *J. Sci. Food Agric.* **101**, 693-702 (2021).
